# Biological Activity of Extracts of Red and Yellow Fruits of *Cornus mas* L.—An In Vitro Evaluation of Antioxidant Activity, Inhibitory Activity against α-Glucosidase, Acetylcholinesterase, and Binding Capacity to Human Serum Albumin

**DOI:** 10.3390/molecules27072244

**Published:** 2022-03-30

**Authors:** Olha Dzydzan, Iryna Brodyak, Paulina Strugała-Danak, Angelika Strach, Alicja Z. Kucharska, Janina Gabrielska, Natalia Sybirna

**Affiliations:** 1 Department of Biochemistry, Ivan Franko National University of Lviv, 4 Hrushevskyi St., 79005 Lviv, Ukraine; olha.dzydzan@lnu.edu.ua (O.D.); iryna.brodyak@lnu.edu.ua (I.B.); nataliya.sybirna@lnu.edu.ua (N.S.); 2 Department of Physics and Biophysics, Wrocław University of Environmental and Life Sciences, C. K. Norwida 25, 50-375 Wrocław, Poland; 269763@student.pwr.edu.pl (A.S.); janina.gabrielska@upwr.edu.pl (J.G.); 3 Department of Fruit, Vegetable and Plant Nutraceutical Technology, Wrocław University of Environmental and Life Sciences, J. Chełmońskiego 37/41, 51-630 Wrocław, Poland; alicja.kucharska@upwr.edu.pl

**Keywords:** *Cornus mas* L., α-glucosidase, acetylcholinesterase, enzyme inhibition, albumin, antioxidant activity

## Abstract

Although extracts are broadly used in order to support the treatment of numerous diseases, only in a limited number of cases is the process of applying and establishing their mechanisms of action scientifically analyzed. Fruits of Cornelian cherry are an abundant source of iridoids, anthocyanins, flavonols and phenolic acids. The aim of the present study was to evaluate the in vitro bioactivity of red and yellow Cornelian cherry fruits’ extracts. The biological potential of extracts, in a broad sense, involved antioxidant activity in relation to phosphatidylcholine liposomes, inhibitory ability against α-glucosidase and acetylcholinesterase enzymes, as well as interactions with human serum albumin. Studies showed that both extracts were more effective in protecting liposome membranes against free radicals produced by AAPH in an aqueous environment due to the fact that they can be better eliminated by the hydrophilic components of the extracts than those produced by UVB radiation. Extracts exhibited inhibitory activity against acetylcholinesterase and α-glucosidase, wherein loganic acid extract showed noncompetitive inhibition of the enzyme. Moreover, extracts binded to albumin mainly through hydrogen bonds and van der Waals forces. Taken together, red and yellow cherry fruits’ extracts exhibit diverse biological properties and can be exploited as a source of natural therapeutic agents.

## 1. Introduction

Cornelian cherry is one of the most valuable species of fruit plants in the family *Cornaceae*. Within the genera *Cornus* there are about 40 species which are widely found in moderate climate zones. In general, these plants are classified as ornamental plants (*Cornus kousa* (Miq.)). One particular species, which bears relatively big fruit and is broadly cultivated by man, is known as Cornelian cherry (*Cornus mas* L.). In recent years, the properties of Cornelian cherry fruit have been broadly analyzed, not only in terms of its taste, but also from a pro-healthy point of view [1,2]. The fruits of the species *Cornus officinalis*, which is common in certain countries in Asia, has been used in traditional cuisine and in the treatment of many pathological conditions for years [3,4]. However, in southern and central Europe and southwest Asia the cultivated species is *Cornus mas* L. The fruit of this species is particularly interesting due to the biologically active components that are present in their composition. The chemical composition of Cornelian cherry fruit is complex and broadly depends on the particular species, as well as the conditions under which this plant is cultivated and both environmental and climatic factors. Quantitative and qualitative component composition of fruits of the species *Cornus mas* L. differs from fruits of the species *Cornus officinalis* [5,6]. In addition, we found a difference in composition between red and yellow fruits of the *Cornus mas* L. Whereas red fruits of the species contain iridoids, anthocyanins, phenolic acids and flavonols, anthocyanins were not detected in the yellow fruit. However, the latter includes more iridoids and phenolic acids [7]. The constituent components of extracts from *Cornus mas* L. exhibit a variety of biological activities, making the extract a promising compound in the treatment of various diseases, especially gastrointestinal and metabolic disorders, diabetes and diabetes-related complications, stomach ulcers, diarrhea, fever, sore throats, urinary tract infections and liver and kidney diseases [8,9]. Moreover, little is known about the biological effects of extract of yellow fruits of *Cornus mas* L., in comparison with extract of red fruits of *Cornus mas* L.

Maintaining physiological blood glucose levels under diabetes is a major issue in the treatment of this disease. It has been shown that under administration of oral hypoglycemic drugs, in particular α-glucosidase inhibitors (acarbose, miglitol, voglibose), it is possible to achieve a state of normoglycemia [10,11]. α-Glucosidase inhibitors are used in diabetes treatment along with other oral hypoglycemic medicine or insulin and may have a higher potential of action and fewer side effects than synthetic drugs [12]. All of these inhibitors affect α-glucosidase (EC 3.2.1.20) which that breaks down complex carbohydrates in the small intestine. The enzyme acts on the final non-reducing α-glucose residues and hydrolyzes α1,4-glycosidic bonds. Catabolism of glycogen, starch and oligosaccharides under α-glucosidase action is accompanied by the release of α-glucose, which is absorbed through the intestinal wall into the blood. Thus, α-glucosidase inhibitors reduce the entry of monosaccharides into the blood and impact on blood glucose level after meals—postprandial hyperglycemia [11].

Uncontrolled increase of blood glucose can lead to the development of oxidative stress. Oxidative stress is one of the main factors contributing to the pathogenesis of many diseases and provokes the development of numerous comorbidities [13,14,15,16]. Free radicals, in particular reactive oxygen species (ROS), play a central role in the oxidative stress progress. ROS modify the structure and functions of proteins, enhance lipid peroxidation (LPO), induce DNA damage and activate signaling pathways and inflammatory processes [17,18]. In particular, the intensification of LPO processes leads to damage of the structural components of cell membranes [19,20]. The phospholipid bilayer of plasma cell membranes is a biological platform for integrated receptors, signaling proteins, antigens, protein transporters of various compounds, adhesive molecules and membrane-bound enzymes. Therefore, its damage leads to increased permeability to hydrophilic molecules and ions, impaired transduction of intracellular signals, metabolism, regulation of gene expression, changes in electrochemical potential, and, as a consequence, cell death [21].

The search for compounds with hypoglycemic, antioxidant and anti-aging properties in order to protect cells against oxidative stress progress and reduce the negative impact of ROS on the lipids of cell membranes is an important issue [19]. Despite the widespread use of synthetic compounds [10], phytotherapy remains a popular subject of scientific research as a source of biologically active compounds with various properties. The principal advantage of natural compounds of plant origin lies in their wide range of action with minimal or no side effects in vivo [7,8,9]. Using drugs based on natural compounds in the early stages of the disease can prevent the development of serious complications or improve the condition of patients in the later stages of a disease progression [14,15,16]. Therefore, plant and herbal medicine can provide significant support for standard treatment.

Many compounds of plant origin that exhibit antioxidant properties have already been studied [22,23,24]. Rational use of plant extracts in treatment will be possible if we understand the mechanism of their action on biological systems at the molecular and cellular levels. One important factor in the biological and pharmacological activity of natural active compounds in the body is their ability to interact with the lipid membrane bilayer of cells [25]. The ability of flavonoids to interact with the polar/nonpolar regions of the lipid bilayer was demonstrated by various experimental approaches [26]. However, it is not fully revealed how complex extracts interact with lipid membranes. In the case of an oxidant, namely potentially one of the most harmful agents, interaction with membrane may be one of the mechanisms involved in the antioxidant action of investigated substances. Promising plant materials that can become the basis of new corrective drugs are fruits of plants of the *Cornaceae* family.

In our previous work, it was found that oral administration of extracts of *Cornus mas* L. fruits to rats with streptozotocin-induced diabetes mellitus led to a decrease in blood glucose level [7]. We suggested that one of the possible mechanisms of blood glucose lowering under the administration of studied extracts might be inhibition of intestinal α-glucosidase activity and, consequently, a decrease in the absorption rate of hydrolysis products from the intestine into the blood.

Therefore, the aim of this in vitro study is to evaluate the inhibitory potential of red and yellow Cornelian cherry (*Cornus mas* L.) fruits’ extracts on the activity of α-glucosidase and acetylcholinesterase enzymes (AChE), determine the ability of extracts binding to human serum albumin and their antioxidant activity under oxidative stress condition, induced by physicochemical factors (UVB radiation and 2,2′-azobis(2-amidinopropane) dihydrochloride radicals (AAPH^●^) on a lipid membrane model.

## 2. Results and Discussion

### 2.1. The Chemical Composition of Red and Yellow Fruits Extracts of Cornus mas L.

**Figure 1:**
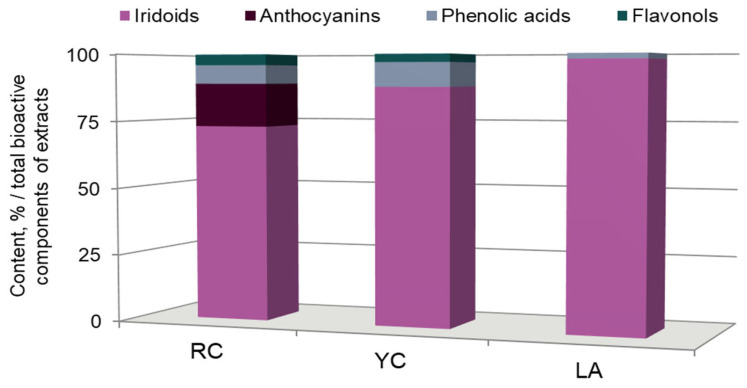
The content (% per 100 g dw) of the compounds of extracts of red (RC) and yellow (YC) fruits of Cornelian cherry, and the purified loganic acid (LA) extract from yellow Cornelian cherry fruits by LC-MS and HPLC-PDA.

This analysis of red and yellow fruits extracts of *Cornus mas* L. has established their qualitative and quantitative composition (Figure 1). A detailed description of the identified compounds in red and yellow fruits extracts of *Cornus mas* L. was included in our previous article [7].

The quantitative and qualitative analysis showed that the red Cornelian cherry fruits’ extract contained 5.7 g per 100 g dry weight (dw) of phenolic compounds, while extract of yellow fruits of Cornelian cherry was identified at the level 2.7 g/100 g dw [7]. Red fruit extract contained iridoids, anthocyanins, phenolic acids and flavonols. In contrast, yellow fruit extract did not contain anthocyanins, but contained 10% more iridoids and only 2% more phenolic acids. Since the main iridoid glycoside of yellow and red fruits of Cornelian cherry is loganic acid, it was also extracted as a separate fraction from the yellow fruits [27]. The obtained fraction was analyzed and it was revealed that the content of loganic acid was 98% (Figure 1).

Phenolic compounds are well known due to their vast pharmacological properties. Many studies have shown a strong and positive correlation between the phenolic compound contents and the antioxidant potential of fruits and vegetables [28]. The aromatic rings are not synthesized in humans and animals, therefore, phenolic compounds are an important component of the diet [29]. The subclasses of phenolic phytochemicals presented in the red fruits of the species *Cornus mas* L. are anthocyanins. Besides the fact that anthocyanins play a role as natural dyes, they also possess various beneficial health effects, including antioxidant and antimicrobial activities [30]. The other constituent components, iridoids and their glycosides, show a wide range of t actions such as cardiovascular, hypoglycemic, hypolipidemic, antioxidant, anti-inflammatory, immunomodulatory action, etc. [8,31,32]. Rational use of plant extracts in treatment will be possible if we understand the mechanism of their action on biological systems at the molecular and cellular levels.

### 2.2. Antioxidant Activity

The study of the antioxidant activity of the extracts from red and yellow fruits of *Cornus mas* L. was based on their ability to protect lipid membrane from oxidation by free radicals. In our experiments, we used two factors inducing oxidation of lipid membrane: UVB radiation and the AAPH compound.

**Figure 2:**
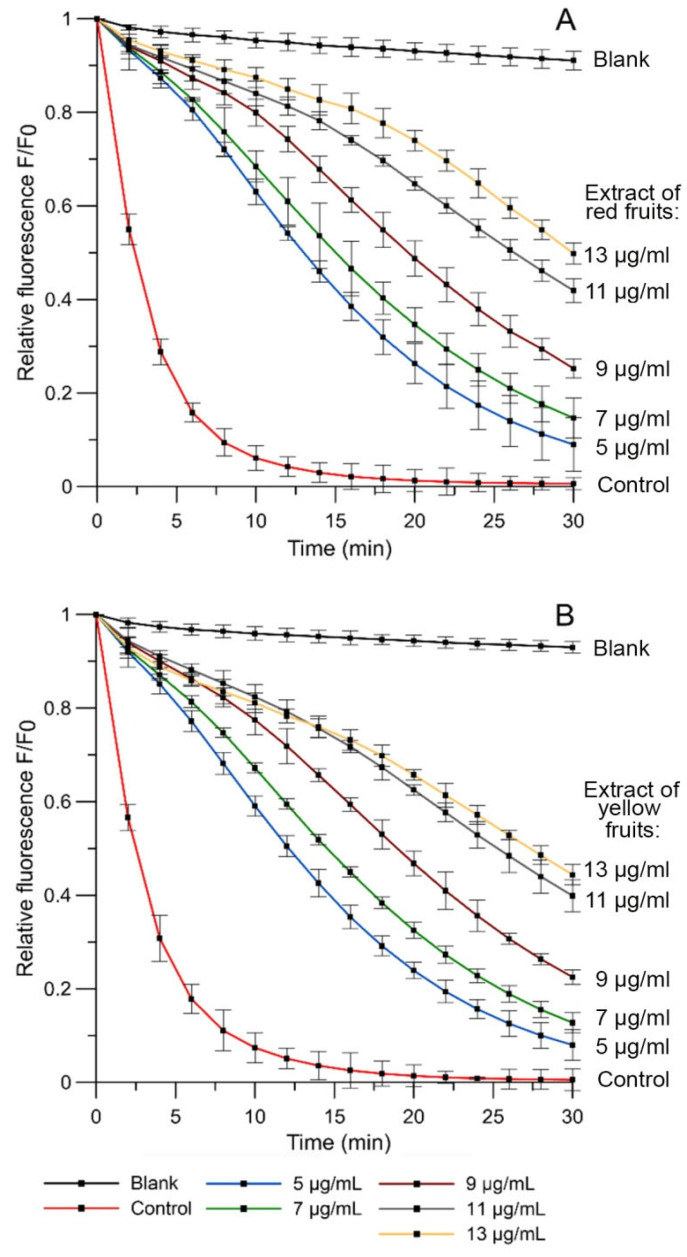
Relative fluorescence intensity of DPH-PA probe as a function of oxidation time for PC liposomes and AAPH^●^ radicals in the presence of extracts from (**A**) red Cornelian cherries and (**B**) yellow Cornelian cherries at five concentrations. The relative change in fluorescence intensity *F*/*F*_0_ is a measure of the degree of lipid peroxidation (*F*_0_, fluorescence intensity in control sample; *F*, fluorescence intensity of samples in the presence of extract).

The kinetic curves of relative fluorescence intensity for the probe 3-[p-(6-phenyl)-1,3,5-hexatrienyl]propionic acid (DPH-PA) in the presence of extracts of red and yellow fruits of *Cornus mas* L. in phosphatidylcholine (PC) liposome membrane are presented in Figure 2. ROS induced by AAPH oxidize the DPH-PA probe, leading to the quenching of its fluorescence. The value of the relative fluorescence intensity of that probe was taken as an indicator of the degree of oxidation of the lipid membrane. It was calculated as the ratio of the fluorescence intensity of the DPH-PA after 30 min of oxidation in the presence of studied extracts, or in the control sample, to the initial intensity value. Bioactive substances contained in the extracts when sweeping away free radicals reduced the decrease in intensity of DPH-PA probe fluorescence. According to the obtained results, the fluorescent intensity increased in a concentration-dependent manner for both extracts of red and yellow fruits of *Cornus mas* L., indicating a decrease in the number of free radicals in the solution. The plot of the relation between the degree of oxidation inhibition versus extracts of red and yellow fruits of *Cornus mas* L. concentrations was the basis to specify the IC_50_ parameter (plots not presented). Parameter of IC_50_ indicates the concentration of an extract that causes 50% reduction of the liposomal oxidation. The values of this parameter are presented in Table 1; e.g., in comparison with L-(+)-ascorbic acid.

We have also investigated the oxidation of lipid membranes exposed to UVB radiation in the presence or absence of the studied extracts. The level of lipid oxidation was determined spectrophotometrically based on the concentration of malondialdehyde (MDA) formed as a result of LPO. According to our data, extracts of red and yellow fruits of *Cornus mas* L. led to a decrease in the value of absorbance at λ = 535 nm of the samples due to a slowdown of the oxidation of the lipid membrane. Since flavonoids by their nature act as unique UV filters [33], it might be assumed that they have a similar effect on lipid membranes exposed to UV radiation. We calculated the percentage inhibition of lipid oxidation for the selected exposure time of 2 h and determined IC_50_ as concentration that reduces oxidation by 50% (Table 1).

We suppose that the antioxidative activity of both extracts is caused by their molecular composition. According to our previous data [7], extracts contained four classes of bioactive compounds such as iridoids, anthocyanins, phenolic acid and flavonols. The studied extracts had a similar component composition relative to the percentages of iridoids (RC: 73.5%; YC: 88.2%), but differed in the content of anthocyanins (RC: 16% vs. YC: 0%). It was shown that the antioxidant capacity of extracts was highly correlated with the total content of polyphenols and flavonoids. Flavonoids, including anthocyanins, possess a broad spectrum of chemical and biological activities, including antioxidant properties. The antioxidant activity of flavonoids depends on the location of functional groups relative to the nuclear structure. The most important determinant for scavenging of ROS and reactive nitrogen species (RNS) is the arrangement of a hydroxyl due to the fact that this group donates hydrogen and an electron to reactive oxygen radicals, stabilizing them. Due to their lower redox potentials, flavonoids (Fl-OH) are thermodynamically able to reduce strongly oxidizing free radicals [34]. However, the IC_50_ values of liposomes oxidation of red and yellow fruits’ extracts did not differ significantly, which may also indicate that the absence of anthocyanins in yellow fruits does not cause a principle decrease in their antioxidant properties. Flavones, and their hydroxylated derivatives, flavonols, are important subgroups of flavonoids. Flavones and catechins proved to be the most powerful flavonoids to protect against ROS [35]. Flavonols were identified in Cornelian cherry extracts from red and yellow fruits by UPLC-ESI-qTOF-MS/MS and HPLC-PDA (Figure 1), suggesting the antioxidant properties of the extracts.

Both extracts were effective in lipid membrane protection against free radicals induced by AAPH^●^. It was shown that the extract of red and yellow fruits of *Cornus mas* L. demonstrated 1,8 and 1,9-fold higher antioxidative activity than L(+)-ascorbic acid (Table 1).

**Table 1:** Antioxidant parameters (IC_50_) for red and yellow Cornelian cherries fruits’ extracts. AAPH^●^ compound and UVB irradiation induce oxidation of PC liposomes.

Inducer Free Radical	Red Cornelian Cherries (μg/mL)	Yellow Cornelian Cherries (μg/mL)	L(+)-Ascorbic Acid (μg/mL)
AAPH	12.60 ± 0.87	12.39 ± 0.96	22.80 ± 2.19 *
UVB	26.48 ± 3.90	26.24 ± 2.11	115.3 ± 2.50 *

* Strugała et al. [36].

In addition, our studies have shown that both extracts are more effective in protecting the lipid membrane against free radicals induced by AAPH^●^ than those produced by UVB radiation. The lipid bilayer is a thin polar membrane made of amphiphilic lipid molecules. UVB radiation can penetrate the entire membrane and generate free radicals in its hydrophobic interior. In return, free radicals produced by AAPH^●^ in an aqueous environment are better eliminated by the hydrophilic components of the extract. The ability of chemical compounds to penetrate into lipid bilayers is a crucial factor in their protection against oxidation. As mentioned before, one of the main components of both extracts are phenolic/polyphenolic compounds. It has been shown that they can inhibit the spread of lipid oxidation by two mechanisms: (1) by intercepting intramembrane radicals and/or (2) increasing membrane fluidity. The principle of the second mechanism is associated with the disorganization of lipid chains and, as a consequence, inhibition of radicals’ propagation. By contrast, the fluidizing of the membrane can promote efficient interaction of antioxidant molecules with lipid radicals [37,38].

### 2.3. Alpha-Glucosidase Inhibition

**Figure 3:**
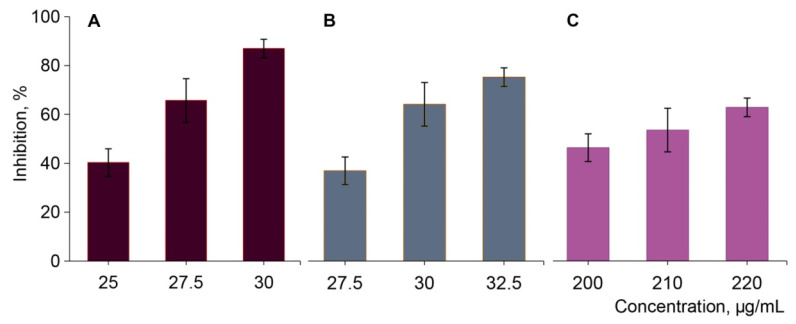
Inhibitory potency of Cornelian cherry extracts from red (**A**) and yellow (**B**) fruits, and the loganic acid (**C**) against α-glucosidase activity. The values are expressed as means ± SEM of triplicate tests.

The impact of the extracts of yellow and red Cornelian cherry fruits and the purified loganic acid extracted from yellow fruits on α-glucosidase activity from *Saccharomyces cerevisiae* was estimated. Our in vitro studies revealed a strong inhibitory effect of Cornelian cherry extracts on α-glucosidase activity. The extracts of red and yellow fruits exhibited the highest inhibitory potential on the enzyme when compared to purified loganic acid extract from Cornelian cherry fruits (Figure 3). The extract of red fruits at a concentration of 25.7 and 30.0 μg/mL possessed a higher inhibitory effect on the activity of α-glucosidase compared with the extract of yellow fruits at the same concentration (Figure 3). The inhibitory activity of loganic acid (IC_50_ 211.56 μg/mL) on α-glucosidase was 8.0 and 7.5 times weaker compared to red and yellow fruits extracts, respectively (Table 2). Therefore, in vitro assays showed stronger inhibition of α-glucosidase by the extract of red fruits of Cornelian cherry in contrast to extract of yellow fruits and its major component: loganic acid, an iridoid glycoside extracted from *Cornus mas* L. fruits.

α-Glucosidase, as one of the enzymes of complex carbohydrates catabolism in the digestive tract, hydrolyzes α-glycosidic bonds of polysaccharides with the release of free glucose. As monosaccharides are absorbed only in the intestinal lumen and transported into the blood [11], α-glucosidase is an important regulatory factor in the stage of glucose absorption in the intestinal mucosa. Lowering the glucose uptake through the inhibition of α-glucosidase in the small intestine is one of the postprandial hyperglycemia treatment strategies of diabetes and other hyperglycemic conditions.

Medicinal plant studies indicate that biologically active substances belonging to the class of flavonols might be potential α-glucosidase inhibitors [39,40]. The observed inhibitory effect of extracts of Cornelian cherry fruits might be a synergic effect of total bioactive compounds present in the extracts. The effective inhibition of α-glucosidase was also proven by anthocyanins-rich extracts of blueberry and blackcurrant examined by Panahi et al. [41]. Therefore, the red and yellow fruits’ extracts of *Cornus mas* L. are natural inhibitors of α-glucosidase activity and can be used in treatment of postprandial hyperglycemia.

**Figure 4:**
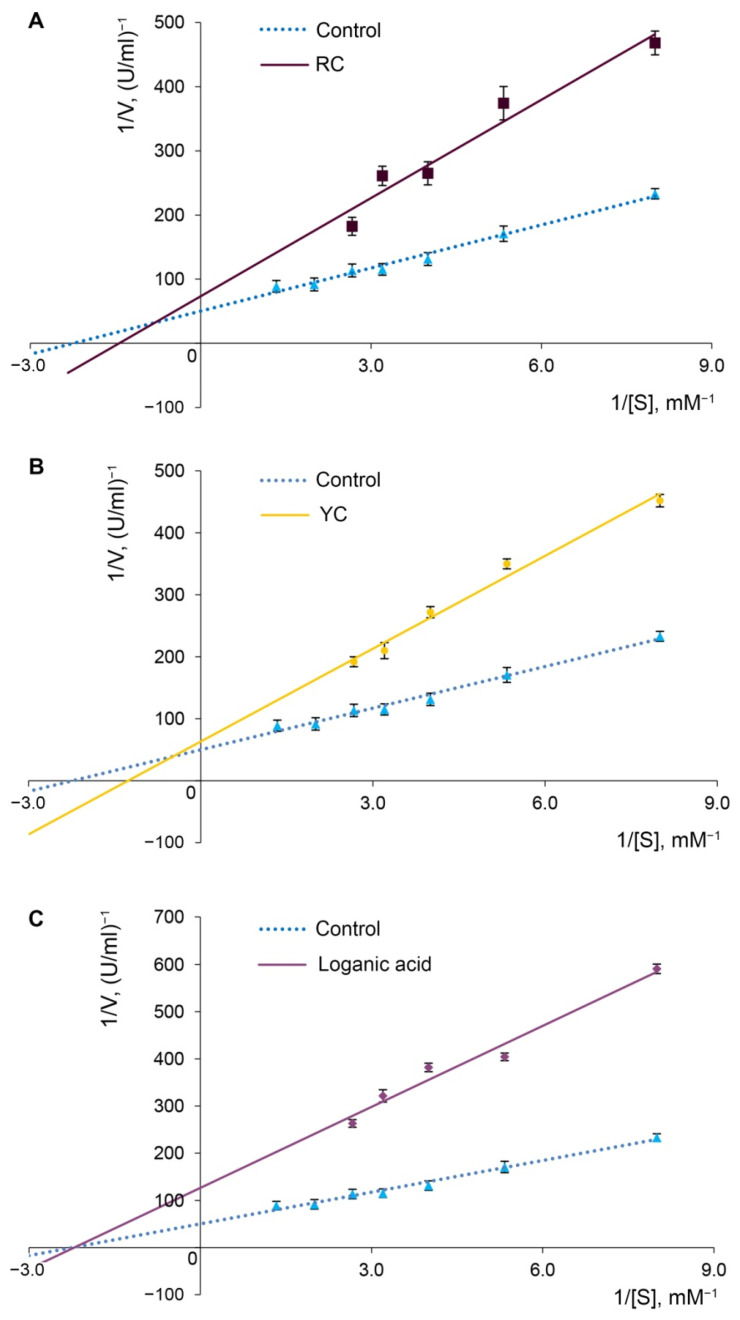
Mode of inhibition of α-glucosidase (Lineweaver–Burk plot) by Cornelian cherry extracts from red (**A**) and yellow (**B**) fruits, and the purified loganic acid (**C**) extract from Cornelian cherry yellow fruits.

The different inhibition potential of α-glucosidase by Cornelian cherry extracts from red and yellow fruits and the purified loganic acid indicate that different inhibition mechanisms are involved. The mode of inhibition of Cornelian cherry fruits’ extracts on α-glucosidase was determined using the Lineweaver–Burk plot, which displayed a mixed inhibition of the enzyme, while loganic acid extract proved to be the noncompetitive inhibitor of α-glucosidase (Figure 4). The non-competitive type of inhibition in the Lineweaver–Burk coordinates indicates that the active components of the extract do not compete with the substrate for binding in the active site of the enzyme. They bind to another site on the enzyme molecule that causes an inhibition of catabolism of complex carbohydrates to monosaccharides [42].

Due to the presence of phytochemicals such as anthocyanins, flavonols and iridoids, extracts of red and yellow fruits of Cornelian cherry showed a distinctive type of inhibition of the studied enzyme compared to loganic acid. Qualitative analysis of extracts revealed that the red fruit extract contains quercetin and kaempferol derivatives, such as quercetin 3-*O*-galactoside, quercetin 3-*O*-glucuronide, quercetin 3-*O*-glucoside, kempferol 3-*O*-galactoside and kaempferol- 3-*O*-glucuronide, and yellow fruit extract—only quercetin derivatives [7]. It is known that anthocyanins, particularly pelargonidin glycosides, and other flavonoids were distinguished as the potent inhibitors of α-glucosidase, much better than the other phenolic molecules [39,43]. The glycosylation in anthocyanidins, presence of double bond in a C-ring and exact distribution of hydroxyl groups on the flavonoid core are chiefly responsible for the action mechanism and inhibitory capacity of polyphenols on α-glucosidase [44]. It was proved that the pelargonidin 3-*O*-robinobioside places into the enzyme binding pocket and forms multiple hydrogen bonds and hydrophobic interactions [39]. While Ning et al. [45] reported that the inhibitory activities of flavonols (quercetin, kaempferol, quercetin-3-*O*-glucuronide and kaempferol-3-*O*-rhamnoside) are probably caused by their easy occupation of the active site of the α-glucosidase, it was also shown that quercetin is one of the strongest α-glucosidase inhibitors [46].

There is not enough available literature to show the inhibitory potential of loganic acid against α-glucosidase. While Szczepaniak et al. [47] established that the high permeability of loganic acid (over 1 × 10^−6^ cm/s) allows the characterization of LA as an extra bioavailable substance, they supposed that loganic acid also might have a structural affinity to enzymes, including those regulating glucose metabolisms [47].

It is worth emphasizing that some research showed that the in vitro gastric digestion process had no negative effect on both the anthocyanins and total antioxidant potential of digested Cornelian cherry extracts [48].

Our previous studies on the antidiabetic effect of extracts of red and yellow Cornelian cherry fruits in vivo showed that extracts were responsible for lowering glucose levels in rats with streptozotocin-induced diabetes, but their mechanism of action remains unclear [7]. We suppose that Cornelian cherry fruit extracts, acting as α-glucosidase inhibitors, are able to reduce glucose absorption in the small intestine and prevent a rise in glucose levels after meals. Therefore, one of the mechanisms of the hypoglycemic effect of extracts may be the inhibition of α-glucosidase activity in the animals’ intestines.

The search for alternative drugs based on medicinal plants, which would be characterized by higher potential, minor side effects and a combined therapeutic effect in the condition of diabetes is extremely important and promising for both medical and biological tasks nowadays. One of the strategies of treatment for diabetes is the inhibition of carbohydrate digestion enzymes activity in the gastrointestinal tract. In that case, extract of the α-glucosidase inhibitor should be taken at the beginning of the main meal to achieve the maximum hypoglycemic effect [11]. It can be assumed that the effectiveness of the extracts in reducing the degree of postprandial hyperglycemia depends on the amount of complex carbohydrates in the diet. However, unlike acarbose (an α-glucosidase inhibitor), extracts of red and yellow fruits of Cornelian cherry may be absorbed into the blood and act not only in the intestine, but show antidiabetic and antioxidant effects in the whole body [7,27].

### 2.4. Acetylcholinesterase Inhibition

It is a well-known fact that there is a link between oxidative stress and the development of various diseases, namely neurodegenerative, diabetes mellitus and cancer. Alzheimer disease (AD) is a neurodegenerative disease which is characterized by memory loss and significant reduction in cognitive functions. AD is one of the most frequently diagnosed types of dimension typical for elderly people. Moreover, it is proved by numerous scientists that the ROS and other free radicals, which are formed and revealed during oxidative stress and which are a consequence of the lack of equilibrium between their production and the ability of the antioxidative system to remove them, are responsible for inducing cellular and molecular abnormalities in AD [49]. As it is suggested by the cholinergic hypothesis, the reduction in acetylcholine (ACh) synthesis leads to AD. This is the underlying cause of elevating acetylcholine by inhibiting acetylcholinesterase (AChE), which, in turn, is responsible for the breakdown of ACh, and which is a significant strategy in developing drugs in AD [50]. Plants and their extracts are excellent resources of AChE inhibitors, and show huge potential to help scientists in novel strategies for the prevention and treatment of these diseases [51].

The result of the AChE inhibitory activity in case of the studied extracts is shown in Table 3. The obtained data proves that extract of red Cornelian cherry is responsible for inhibiting AChE activity by 70.1% at a concentration of 1 mg/mL, while extract of yellow Cornelian cherry is responsible for inhibiting the AChE activity by 58.4% under the same conditions (Table 3). The above-mentioned data proves that the anti-Alzheimer’s potential of extract of red fruits of *Cornus mas* L. was greater in inhibiting AChE (about 1.2 times) while compared with the extract from yellow fruits of *Cornus mas* L. However, the inhibiting properties of both extracts were weaker towards AChE compared to neostigmine (Table 3). The higher potential of extract of red Cornelian cherry to inhibit AChE may be explained by the presence of anthocyanins, which had a total concentration in the RC extract that was estimated as higher than 3.07 g/100 g dw. Several recent studies have supported the view that anthocyanins show AChE activity and play a neuroprotective role [52,53]. Going beyond in vitro evidence, several reports focused on assessing the efficacy of anthocyanins in various animal models of AD have been published in recent years. To illustrate, in vivo studies showed that the anthocyanin-rich extract from wild blueberries introduced intraperitoneally at 60 mg/kg bw and displayed high AChE inhibitory activity in mice [54]. Studies conducted on elderly people who were at risk of dementia also delivered promising data in reference to anthocyanin consumption. The dietary introduction of blueberries and Concord grape juice, which are rich in anthocyanins, significantly improved mild memory impairment in treated individuals [55]. Moreover, another study also proved that a 12-week dietary intervention with anthocyanin-rich cherry juice significantly improved short and long-term memory in a group of elderly adults who suffered from both mild to moderate dementia [56,57].

The ability to inhibit AChE in the case of extracts from red fruits of *Cornus mas* L. may be caused not only by anthocyanin, but also by other phenolic compounds which are present in the extract. There are numerous reports which prove an affirmative effect of phenolic compounds in neurodegenerative diseases. Orhan et al. conducted a screening study of various phenolic acids (chlorogenic, caffeic, gallic and quinicacids), as well as flavonoid derivatives (naringin, quercetin, luteolin-7-*O*-rutinoside, kaempferol-3-*O*-galactoside, apigenin, biochanin and silibinin) in order to test their inhibitory activities against AChE and butyrylcholinesterase [58]. In this group of compounds, only quercetin showed a huge ability to inhibit AChE (approximately 76%) in the concentration of 1 mg/mL, whereas genistein, luteolin-7-*O*-rutinoside and silibinin showed activity in relation to butyrylcholinesterase. Sheng et al. [59] designed a new series of flavonoid derivatives as potent AChE inhibitors and observed that the vast majority of them showed greater inhibitory activities to AChE than a drug called rivastigmine. The most potent inhibitor, namely isoflavone derivative, showed greater impact towards AChE, superior 389-fold to donepezil [59].

Whereas phenolic compounds are well-known for their potent antioxidant effects, the plant extracts, including used in this study red and yellow fruits of Cornelian cherries, also have significant inhibitory properties towards AChE and may be considered as promising in new therapies.

### 2.5. Binding to Human Serum Albumin

The affinity between chemical compounds and proteins is also important in order to understand the biological activity of the studied extracts. Thus, Codorniu-Hernández et al. [60] in their work showed that there is an affinity between flavonoids and amino acid residues of some proteins, including the important protein, human serum albumin (HSA). Albumin is the most important carrier of exogenous and endogenous molecules in human plasma, like hormones, enzymes, medicines and toxins. HSA represents approximately 50% of the total protein content in healthy humans [61]. Understanding and characterizing the interaction of compounds with HSA has attracted much research interest for decades. The nature of these bindings affects pharmacokinetics, pharmacodynamics and therapeutic efficacy and is a key aspect during the process of drug delivery and designing [62]. The impact of such drugs on the overall conformation, stability and function of HSA is also important.

We analyzed the emission spectra of HSA fluorescence in the absence or presence of the tested extracts in order to demonstrate the binding of the compounds of extracts to HSA.

**Figure 5:**
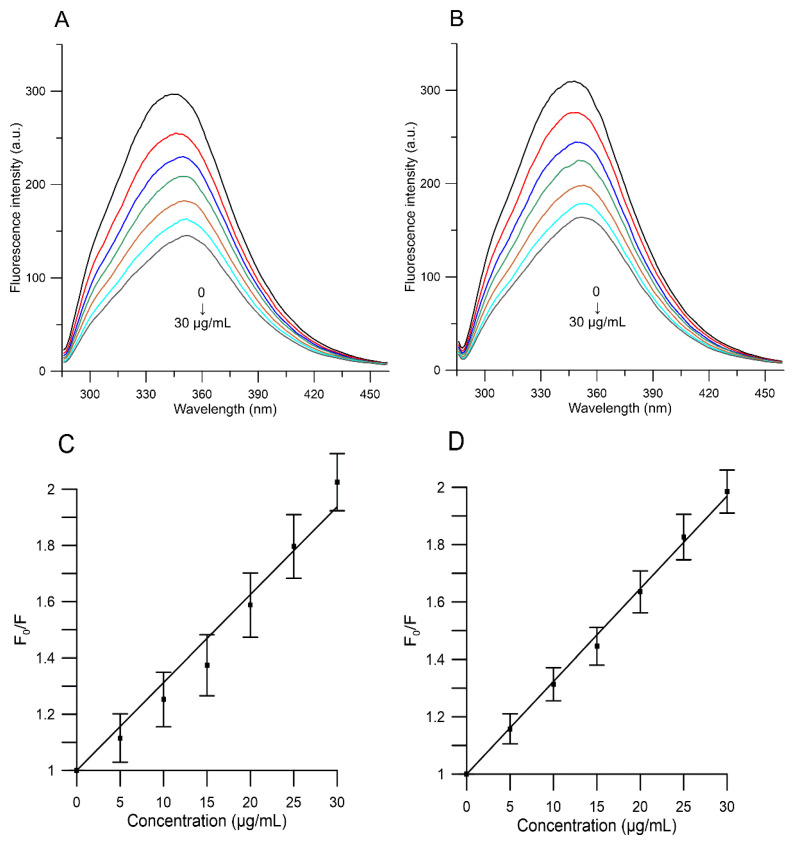
Emission spectra of HSA in the presence of various concentrations of red (**A**) and yellow (**B**) Cornelian cherries fruits’ extracts and Sterne Volmer plots of *F*_0_/*F* against concentration of red (**C**) and yellow (D) Cornelian cherries extracts. Control is marked black and consecutive spectra of the extracts (marked color) are in the following concentrations 5, 10, 15, 20, 25 and 30 µg/mL (HSA = 1.5 × 10^−5^ M, T = 310 K).

Figure 5 shows that the intensity of HSA fluorescence decreased with an increase in the concentration of the extracts (5, 10, 15, 20, 25 and 30 µg/mL) from both red and yellow fruits of Cornelian cherry, which indicated that the compounds quench the internal fluorescence of HSA.

We used the Stern–Volmer equation [63] in order to analyze the fluorescence quenching mechanism induced by the tested extracts at different temperatures (295, 300, 305, 310 and 315 K):

$$\frac{F_{0}}{F}=1+K_{q}\tau_{0}\left[Q\right]=1+K_{SV}\left[Q\right],\;$$

where: *F*_0_ and *F* refer to HSA fluorescence intensities before and after adding quencher, *k_q_* refers to a bimolecular quenching constant, *τ_0_* refers to a quenching rate constant and average lifetime (10^−8^ s^−1^), [*Q*] refers to the quencher concentration and *K_SV_* refers to the Stern–Volmer quenching constant (*K_SV_ = k_q_τ*_0_).

There are two types of fluorescence quenching: static (contact quenching) or dynamic (collisional quenching). Static quenching is when the quencher, being in direct contact with the fluorophore, results in complete absence of its radiation. The formed non-fluorescent ground-state complex has a unique absorption spectrum. Dynamic quenching is determined by the collision of the quencher with fluorophore and does not impact on the probe’s absorption spectrum [64].

One of the factors that helps to distinguish the static and dynamic quenching is their different dependence on temperature. The increase in temperature results in faster diffusion. Thus, higher temperature leads to larger amounts of collisional quenching and smaller amounts of static quenching [63].

In line with our results (Table 4), *K_SV_* of the extracts does evidently decrease with increasing temperature, indicating that the main fluorescence quenching mechanism is static.

We assume that the mechanism of HSA quenching by both extracts from Cornelian cherry is based on the formation of a complex. It is assumed that the quenching process is limited by diffusion. This means that the molecule (HSA) and the quencher are much less likely to approach each other than interact when they actually meet. In particular, quenching will occur more frequently if the quencher and the molecule should meet more frequently [65].

The binding affinity of any drugs to serum albumin is one of the major factors that determine the pharmacokinetics. Serum albumin is one of the principal carriers, binding to which increases the apparent solubility of hydrophobic substances in the plasma [66]. Several other parameters like apparent binding constants (*K_b_*) and number of binding sites (*n*) were used to evaluate the interaction process. The higher binding constant values show the stronger binding interaction between substance and HSA, indicating that the drug will be highly bound in blood plasma in vivo. The values of n and *K_b_* were obtained by plotting:

$$log\left(\frac{F_{0}-F}{F}\right)=logK_{b}+nlog\left[Q\right],\;$$

where, *F_0_* and *F* indicate the magnitude of fluorescence intensity in the absence and presence of quencher (extract), *K_b_* is the binding constant and *n* is the number of binding sites. From linear plot of *log (F*_0_ *− F)/F* versus *log* [*Q*], the values of *K_b_* were obtained from the intercept.

**Figure 6:**
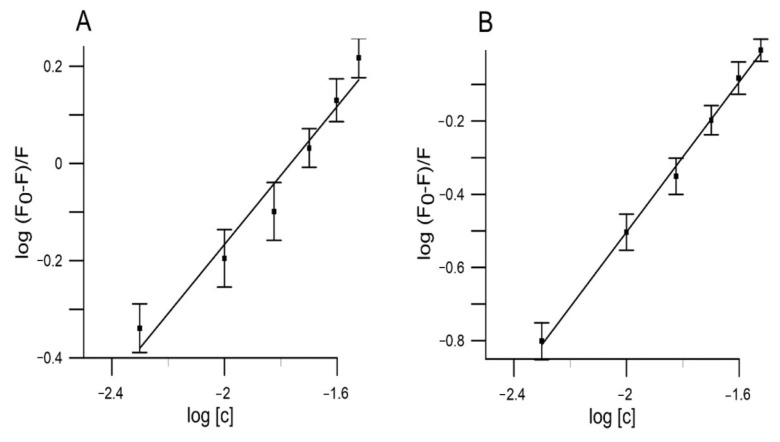
The plots of *log*(*F*_0_ − *F*)/*F* versus *log*(*c*): (**A**) red Cornelian cherries fruits extract, (**B**) yellow Cornelian cherries fruits extract (HSA = 1.5 × 10^−5^ M, T = 310 K, λ_ex_ = 280 nm, λ_em_ = 345 nm).

The results for studied extracts at five different temperatures (295, 300, 305, 310 and 315 K) are given in Table 4 and Figure 6. According to our results, the values of *K_b_* for the extracts of red and yellow fruits of *Cornus mas* L. decreased with increasing temperature. The data indicate that the complex of the studied extracts with HSA became unstable when the temperature went up.

Numerous research studies show that drugs bind to sites of high affinity with typical association constants in the range of 10^4^–10^6^ M^−1^. The binding affinity of both of our studied extracts is within these limits.

One of the components of the extracts is flavonoids. These compounds were found to bind in the subdomain IIA of HSA, with the exception of flavanones. At physiological pH, ring B of flavonoids has a partial negative charge, while rings A and C form a hydrophobic part, creating ideal conditions for flavonoid binding [67]. However, the extract of red fruits of *Cornus mas* L. showed a stronger affinity for binding to HSA (*K_b_* = 3.705 × 10^4^ at the lowest tested temperature of 295 K) than the extract from yellow fruits (*K_b_* = 1.752 × 10^4^ at the lowest tested temperature of 295 K). Such data may be due to the fact that the extract of red fruits contains anthocyanins, one of the members of the flavonoid group of phytochemicals, which are absent in the composition of yellow fruits of Cornelian cherry.

In addition, it was shown [68,69] that glycosylation of flavonoids creates a steric barrier in the binding pocket and increases the polarity of the molecule, thereby reducing the ability of flavonoids to penetrate tryptophan-rich hydrophobic internal regions of HSA, and thus significantly weakening the binding affinity. According to our previous investigation [7], the extracts of red and yellow fruits of *Cornus mas* L. contain quercetin 3-*O*-galactoside, quercetin 3-*O*-glucuronide, quercetin 3-*O*-glucoside and kaempferol 3-*O*-galactoside. Only in the composition of red fruits were identified three monoglucosides (delphinidin 3-*O*-galactoside, cyanidin 3-*O*-galactoside, pelargonidin 3-*O*-galactoside) and two diglucosides (cyanidin 3-*O*-robinobioside, pelargonidin 3-*O*-robinobioside). Such modification of flavonoids may also affect binding affinity to HSA. It was also shown, based on the rutin investigation, that the glycosidic form of quercetin has a lower affinity for BSA than quercetin due to loss of conformational flexibility by glycosylation [70]. However, it has been shown that the replacement of the functional hydroxyl group in the A ring is a more critical factor for flavonoid interaction with albumin than are substituents on the hydroxyl group in the C ring. Replacement of the 3-OH group in the C ring caused a decrease in affinity to BSA by an order of magnitude, while the binding affinities decreased by two orders of magnitude in case of replacement of the 7-OH group [71].

Instead, anthocyanidins were found to bind to HSA with greater affinity compared to quercetin and kaempferol [72] due to the greater number of hydroxyl groups in the B ring and the presence of a positive charge on the C ring. Such positive charge on the C-ring can interact with protein negative charges and stabilize anthocyanidin–HSA complexes, which explains its greater stability over quercetin and kaempferol complexes [66].

Another important component of the extracts is iridoids, which belong to the class of monoterpenoids based on a cyclopentan-[C]-pyran skeleton. There are few studies that reveal the peculiarities of HSA and iridoids interaction. Beema Shafreen et al. [73] have shown decreasing of the fluorescence intensity of measured peaks in HSA during interaction with some monoterpenes. Furthermore, we found that the one molecule of HSA is associated with a single molecule of both of the extracts at five different temperatures (*n* = 1.09–0.85 for extract from yellow fruits and *n* = 1.05–0.83 for extract from red fruits of Cornelian cherry).

The interaction forces involved in binding of drugs with biomacromolecules are usually classified into hydrogen-bond formation, van der Waals, electrostatic and hydrophobic forces [74]. Thermodynamic parameters such as free energy change (Δ*G*), enthalpy change (Δ*H*) and entropy change (Δ*S*) provide valuable information in understanding the model of interaction. The positive values of Δ*H* and Δ*S* suggested that hydrophobic interactions were the major contributing forces in binding between molecules. On the other hand, the negative values of Δ*H* and Δ*S* indicate the presence of van der Waals force or hydrogen bonds. The positive value of Δ*S* and negative value of Δ*H* demonstrated the significant role of electrostatic interactions.

**Figure 7:**
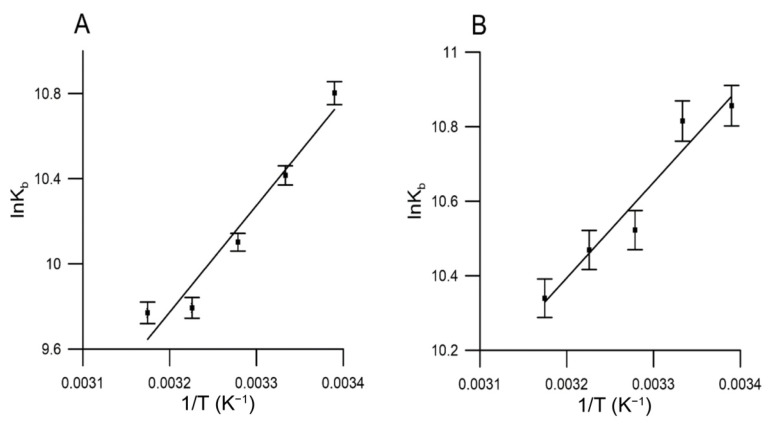
Van’t Hoff plot for temperature dependence of *K_b_*. Obtained from HSA fluorescence quenching by red (**A**) and yellow (**B**) Cornelian cherries fruits extracts.

The thermodynamic parameters can be calculated by the Van’t Hoff’s equation [74]:

$$lnK_{b}=\frac{-\Delta H}{RT}+\frac{\Delta S}{R}\;\;\Delta G=\Delta H-T\Delta S=-RTlnK_{b}\;$$

where $K_{b}$ represents the binding constant at its corresponding temperature and *R* the gas constant. Δ*G* can be determined using the Van’t Hoff plot (Figure 7), where Δ*H* is the slope and Δ*S* the intercept.

Table 4 and Figure 7 contain the obtained values for Δ*G*, Δ*H* and Δ*S*. Based on the received data, we can assume the spontaneous nature of the binding interactions for both extracts with HSA, as evidenced by the negative values of Δ*G*. In addition, the negative Δ*H* and Δ*S* values suggest that extracts from red and yellow fruits of *Cornus mas* L. bind to HSA mainly through hydrogen bonds and van der Waals forces.

## 3. Materials and Methods

### 3.1. Materials

Acetonitrile for liquid chromatography–mass spectrometry was purchased from POCh (Gliwice, Poland). Loganic acid (LA), loganin (L), sweroside (S), cyanidin 3-*O*-glucoside (Cy glc), 5-*O*-caffeoylquinic acid (5-CQA, chlorogenic acid), caffeic acid (CA), *p*-coumaric acid (*p*-CoA), ellagic acid (EA), quercetin 3-*O*-glucoside (Q-glc) and kaempferol 3-*O*-glucoside (Kf-glc) were purchased from Extrasynthese (Lyon Nord, France) and *trans*-caftaric acid from Cayman Chemical Company (Michigan, EUA, Ann Arbor, MI, USA). Acetylthiocholine iodide, DTNB (5,5′-dithiobis(2-nitrobenzoic acid)), acetylcholinesterase (AChE) from Electrophorus electricus, type VI-S, neostigmine bromide, 2,2′-azobis(2-amidinopropane) dihydrochloride (AAPH), human serum albumin (HSA), (lyophilized powder, essentially fatty acid free), L-α-phosphatidylcholine (from egg yolk), *p*-Nitrophenyl, α-D-glucopyranoside (*p*NPG) (N1377-1G), acarbose and α-glucosidase from *Saccharomyces cerevisiae* (EC 3.2.1.20) (G5003-100UN) were purchased from Sigma-Aldrich Co., Ltd. (St Louis, MO, USA). The probe 3-[p-(6-phenyl)-1,3,5-hexatrienyl]propionic acid (DPH-PA) was purchased from Molecular Probes (Eugene, OR, USA). All other chemicals and reagents used in this study were of analytical grade.

### 3.2. Plant Materials and Preparation of Cornelian Cherry Extracts

Cornelian cherry fruits (*Cornus mas* L.) from cultivars (“Yantarnyi” and “Flava” (yellow color) and “Podolski” (red color) were harvested in the Arboretum in Bolestraszyce, near Przemyśl, Poland (Subcarpathian Voivodeship, south-eastern Poland). The plant materials were authenticated by Prof. Jakub Dolatowski (the Arboretum and Institute of Physiography in Bolestraszyce, Przemyśl, Poland), and adequate voucher specimens (“Yantarnyi”–BDPA 14131; “Flava”–BDPA 8795; “Podolski”–BDPA 10462) were deposited at the Herbariums of Arboretum in Bolestraszyce, Poland. Fruits were harvested in August and September 2016 and immediately frozen at −20 °C. Extracts of red and yellow fruits of *Cornus mas* L. were prepared at the Department of Fruit, Vegetable and Plant Nutraceutical Technology in Wrocław University of Environmental and Life Science (Poland), in accordance with Dzydzan et al. [7]. Frozen ripe Cornelian cherry fruits were shredded and heated for 5 min at 95 °C using a Thermomix (Vorwerk, Wuppertal, Germany). After cooling down to 40 °C, the pulp was depectinized at 50 °C for 2 h (0.5 mL of Pectinex BE XXL (Novozymes A/S, Bagsvaerd, Denmark) per 1 kg). The juice was obtained in a laboratory hydraulic press (SRSE, Warsaw, Poland) and after filtering, it was run through an Amberlite XAD-16 resin column (Rohm and Haas, Chauny CEDEX, France). Impurities were washed off with distilled water, while polyphenols and iridoids were eluted with 80% ethanol. The eluate was concentrated under vacuum at 40 °C. The solvent was evaporated (Rotavapor, Unipan, Warsaw, Poland) and lyophilized (Alpha 1–4 LSC, Christ, Germany).

### 3.3. Identification of Compounds by Liquid Chromatography-Mass Spectrometry (LC-MS)

The UPLC-qTOF-MS/MS method was described earlier [7]. The identification of compounds was performed using the Acquity ultra-performance liquid chromatography (UPLC) system, coupled with a quadrupole-time of flight (Q-TOF) MS instrument (UPLC/Synapt Q-TOF MS, Waters Corp., Milford, MA, USA), with an electrospray ionization (ESI) source. The separation was achieved on an Acquity BEH C18 column (100 mm × 2.1 mm i.d., 1.7 µm; Waters). Iridoids, phenolic acids and flavonols were explored in the negative mode, while anthocyanins were explored in the positive mode before and after fragmentation.

### 3.4. Determination of Compounds by HPLC

The HPLC-PDA method was described earlier [7]. The quantification analysis was performed using a Dionex system (Germering, Germany), equipped with the diode array detector model Ultimate 3000, quaternary pump LPG-3400A, autosampler EWPS-3000SI, thermostated column compartment TCC-3000SD, and controlled by Chromeleon v.6.8 software (Thermo Scientific Dionex, Sunnyvale, CA, USA). The Cadenza Imtakt column CD-C18 (75 × 4.6 mm, 5 µm) was used. Iridoids were detected at λ = 245 nm, anthocyanins at λ = 520 nm, phenolic acids at λ = 320 nm, ellagic acid at λ = 254 nm and flavonols at λ = 360 nm. Iridoids were expressed as loganic acid or loganin, anthocyanins as cyanidin 3-*O*-glucoside, phenolic acid as 5-*O*-caffeoylquinic acid or *p*-coumaric acid, *trans*-caftaric acid, or ellagic acid, flavonols as quercetin 3-*O*-glucoside or kaempferol 3-*O*-glucoside. The results were expressed as % per 100 g dry weight (dw).

### 3.5. Liposome Preparation

Small unilamellar liposomes (SUVs) consisted of phosphatidylcholine (PC) were prepared in accordance with the methodology described by Strugała et al. [75] and in the work of Gabrielska and Oszmiański [76]. The lipids were dissolved in chloroform, evaporated to dryness under nitrogen and under vacuum for another 60 min. Subsequently, a phosphate buffer (pH 7.4) was added and the sample was vortexed to obtain multilamellar vesicles. Then SUVs were formed using a 20 kHz sonicator for 15 min. The final concentration of lipids in the vesicle suspension was 0.1 mg/mL to determine the antioxidative activity in fluorometric studies and 0.3 mg/mL in thiobarbituric acid reactive substance (TBARS) test.

### 3.6. Liposome Oxidation Assay–Spectrophotometric Method

The experiment was described previously [36,77]. In short, peroxidation of PC liposome lipids was initiated with ultraviolet radiation using a bactericidal UVB lamp. Peroxidation was measured as the TBARS level. The percentage of PC liposome oxidation inhibition was calculated using the formula:

$$\%\;\mathrm{inhibition}=\frac{C_{C}\;-C_{E}}{C_{C}}\;\times \;100\%,$$

where *C_C_* refers to the concentration of malondialdehyde (MDA) in a sample without extract (control) and *C_E_* refers to the concentration of MDA in a sample with the appropriate extract added, measured at λ = 535 nm. All determinations were performed with three independent preparations (*n* = 3) using a Cary 300 Varian spectrophotometer.

### 3.7. Liposome Oxidation Assay–Fluorometric Method

Antioxidant activities of extracts of red and yellow fruits of *Cornus mas* L. were determined using a fluorometric method described earlier by Strugała et al. [78]. In brief, the studies were carried out on PC liposomes, which contained the fluorescent probe DPH-PA. Use was made of the relationship between DPH-PA fluorescence intensity and concentration of free radicals. The probe’s fluorescence decreased with its rising oxidation caused by free radicals, supplied by AAPH^●^. Molecules of this compound underwent thermal decomposition into two free radicals each [79]. The value of relative intensity of DPH-PA fluorescence was adopted as a measure of the degree of lipid membrane oxidation. It was calculated as a ratio of fluorescence intensity after 30 min of oxidation in the presence of antioxidants to the initial value of the intensity. The studied polyphenolic compounds scavenged free radicals and thus caused a lower rate of DPH-PA fluorescence decrease. PC liposomes at a concentration of 0.1 mg/mL in phosphate buffer (pH 7.4) were used, incubated for 0.5 h in darkness with addition of DPH-PA probe (stock solutions were prepared in *N,N*-dimethylformamide (DMF)). Concentration of DMF in a sample did not exceed 0.16%. The wavelengths of excitation and emission for the probe were as follows: λ_ex_ = 355 nm, λ_em_ = 430 nm. Oxidation was initiated just before measurement, using AAPH at a concentration of 1 M at 37 °C (control sample), or in the presence of test substances (80% ethanolic extract from fruits of *Cornus mas* L.). The measurements were conducted with a Cary Eclipse fluorimeter. The percentage inhibition of lipid oxidation was calculated on the basis of the following formula:

$$\%\;inhibition=\frac{\left(F_{E\;}-F_{C}\right)}{\left(F_{B}-F_{C}\right)}\times 100\%,$$

where *F_E_* is relative fluorescence of probe oxidized with AAPH^●^ in the presence of extract, *F_C_* is relative fluorescence of control sample oxidized with AAPH^●^ without extract, *F_B_* is relative fluorescence of the blank sample (not oxidized by AAPH^●^ and without extract).

### 3.8. α-Glucosidase Inhibitory Assay

The effect of the extracts on α-glucosidase activity was determined according to the method described by Kim et al. [11,80] using α-glucosidase from *Saccharomyces cerevisiae*. The substrate solution *p*-nitrophenyl glucopyranoside (*p*NPG) was prepared in 0.1 M phosphate buffer (pH 6.9). 100 µL of α-glucosidase (1 U/mL) with 50 µL of the extracts of different concentrations (0.02–0.20 mg/mL in distilled water) or acarbose (positive control) was preincubated at 37 °C for 10 min. Then 50 µL of 3 mM *p*NPG as a substrate dissolved in 0.1 M phosphate buffer (pH 6.9) was added to start the reaction. The reaction mixture was incubated at 37 °C for 20 min and stopped by adding 2 mL of 0.1 M Na_2_CO_3_. The α-glucosidase activity was determined by measuring the yellow-colored reaction product (*p*-nitrophenol) from *p*NPG, reading the absorbance (A) at λ = 405 nm. The results were expressed as a percentage of the blank control. The control (absorbance of total enzyme activity) included all reagents except the extracts or acarbose. The percentage of inhibition was calculated according to the following equation:

$$\%\;inhibition\;activity=\frac{A_{c}-A_{s}}{A_{s}}\times 100\%,$$

where *A_c_* is the absorbance of the control (100% enzyme activity) and *A_s_* is the absorbance of the tested sample (extracts of *Cornus mas* L. or acarbose). Concentrations of the extracts resulting in 50% inhibition of the enzyme activity (IC_50_) were determined graphically.

### 3.9. Determination of α-Glucosidase Inhibition Type

The mode of inhibition of α-glucosidase by the extracts of red and yellow fruits of Cornelian cherries and loganic acid was determined according to the method described by Ali et al. [81] with slight modifications. The reaction mix contains 100 µL α-glucosidase (0.1 U/mL) and 50 µL substrate solution (*p*NPG) at different concentrations (0.25, 0.5, 0.75, 1.0, 1.25, 1.5, 2.0 and 3.0 mM) in the presence or absence of extracts of *Cornus mas* L. and loganic acid extract. The reaction mixture was incubated at 37 °C for 20 min and stopped by adding 150 µL of 0.2 M Na_2_CO_3_. The amount of reducing sugars released was determined spectrophotometrically using a p-nitrophenol standard curve and converted to reaction velocities. A double reciprocal plot (*1*/*V* versus *1*/[*S*]) where *V* is reaction velocity and [S] is substrate concentration was plotted. The type (mode) of inhibition of the extracts on α-glucosidase activity was determined by analysis of the double reciprocal (Lineweaver–Burk) plot using Michaelis–Menten kinetics [82].

### 3.10. Acetylcholinesterase Inhibition Assay

Acetylcholinesterase activity was determined by the Ellman method [83] and Jin et al. [84] with slight modification. Briefly, the assay was carried out on a 96-well plate, with each well containing 140 µL 0.1 M phosphate buffer (pH 8.0), 10 µL of stock solution of extract and 10 µL AChE (1 U/mL). The plate was incubated for 10 min at 25 °C. After incubation, 10 μL of 10 mM DTNB was added to the reaction mixture. Next, the reaction was initiated by the addition of 10 μL of 14 mM acetylthiocholine iodide. The plate was shaken for 1 min and finally 20 µL of 5% SDS was added to stop the reaction. Control wells containing the same composition but without extract (10 µL 70% ethanol) were included. Absorbance at λ = 412 nm was recorded using plate reader (EPOCH, Bio Tech) after 10 min incubation. All the reactions were performed in five repetitions. The percent of AChE inhibition was calculated as follows:

$$\%\;inhibition=\frac{A_{C}-A_{E}}{A_{C}}\times 100\%,$$

where: *A_E_* is the absorbance of the sample containing extract and *A_C_* is the absorbance of the control sample.

### 3.11. Binding to Human Serum Albumin

Analysis of extracts from fruits of *Cornus mas* L. interaction with human serum albumin (HSA) was performed according to the procedure described by Strugała et al. [77]. The fluorescence measurements were performed on a Cary Eclipse fluorimeter equipped with 1.0 cm quartz cells and a thermostat bath. All quenching experiments were performed at 295, 300, 305, 310 and 315 K. The HSA was prepared in phosphate buffer solution (pH 7.4) in final concentration 1.5 × 10^−^^5^ M. The excitation wavelength was set at λ = 280 nm, and the emission spectra were read in the wavelength range (285–460) nm. Our method consisted of tracking the quenching of natural HSA fluorescence caused by extracts from red and yellow fruits of *Cornus mas* L. (final concentrations 5, 10, 15, 20, 25 and 30 µg/mL) added successively. The stock solutions of extracts from fruits of *Cornus mas* L. were prepared by dissolving in 80% ethanol. The control sample contained an appropriate amount of 80% ethanol. The experiment was performed in three independent replicates (*n* = 3).

### 3.12. Statistical Analysis

The results were expressed as mean ± standard error of the mean (M ± SEM). Analysis of variance (ANOVA) followed by Tukey’s post hoc multiple comparison test was used for data analysis and performed in GraphPad Prism 8.0 (San Diego, CA, USA). Differences between the groups were considered statistically significant at *p* < 0.05.

## 4. Conclusions

In vitro bioactivities assays showed that both extracts of red and yellow fruits of Cornelian cherries have a stronger inhibitory potential of α-glucosidase activity than its major component—iridoid glycoside loganic acid. To illustrate, the IC_50_ values were between 25.68–28.46 μg/mL for red and yellow fruits extracts and 211.56 μg/mL for loganic acid. Whereas extracts of red and yellow fruits of Cornelian cherries showed mixed inhibition of enzymes, loganic acid extract showed the noncompetitive inhibition of α-glucosidase. Therefore, extracts of fruits of Cornelian cherries are natural inhibitors of α-glucosidase activity and can be used in order to prevent and treat postprandial hyperglycemia. In addition, the bioactive components of extracts from red and yellow fruits of Cornelian cherries have significant inhibition abilities on acetylcholinesterase and may be considered as promising in new therapies which are focused on neurodegenerative diseases.

Both red and yellow Cornelian cherry fruits’ extracts possess the antioxidant activity in order to protect lipid membranes against free radicals induced by AAPH with the IC_50_ value of 12.4–12.6 μg/mL and by UVB irradiation with the IC_50_ value of 26.2–26.5 μg/mL, which was 1.8- and 4.4-folds higher than that of control L(+)-ascorbic acid, respectively. This indicates that the absence of anthocyanins in yellow fruit does not cause a significant decrease in antioxidant properties of the tested extract.

The extract of red fruits of *Cornus mas* L. showed a stronger affinity for binding to HSA at the temperature of 295 K (*K_b_* = 3.705 × 10^4^) than the extract of yellow fruits (*K_b_* = 1.752 × 10^4^). In addition, we found that single molecules of both extracts from red and yellow fruits of *Cornus mas* L. are associated with a limited number of HSA molecules, mainly through hydrogen bonds and van der Waals forces.

The results obtained in the current study are the first step in order to explain the mechanism of extracts interaction with biological systems and are fundamental in order to search for both novel and effective drugs which are of great pharmaceutical importance. Red and yellow Cornelian cherry fruit extracts may be used as effective compounds for preventing, ameliorating and delaying pathological changes which are responsible for progression of diseases, including diabetes mellitus and Alzheimer’s disease.

## Figures and Tables

**Table 2:** IC_50_ values for α-glucosidase inhibitory potential of Cornelian cherry extracts from red (RC) and yellow (YC) fruits, and the purified loganic acid (LA) extract from Cornelian cherry fruits.

Extracts	Inhibitory Potency of Cornelian Cherry Extracts (IC_50_, µg/mL)
RC	25.68 ± 0.37
YC	28.46 ± 0.36 *
LA	211.56 ± 3.84 **
Acarbose ^#^	5.68 × 10^3^

^#^ Acarbose was used as a reference inhibitor. The values are expressed as means ± SEM of triplicate tests. * *p* < 0.05 compared to RC, ** *p* < 0.001 compared to RC & YC.

**Table 3:** Acetylcholinesterase inhibitory activities of extracts from red (RC) and yellow (YC) fruits of *Cornus mas* L.

Extracts	Concentration (mg/mL)	Inhibition (%)
RC	1.0	70.1 ± 5.0 **
YC	1.0	58.4 ± 0.4 *
Neostigmine ^#^	5.1 × 10^−5^	50.0 ± 3.3

^#^ Neostigmine was used as a reference inhibitor. Compared to neostigmine: * *p* < 0.05, ** *p* < 0.001.

**Table 4:** The Stern–Volmer quenching constants (*K_sv_*), binding constants (*K_b_*) values and thermodynamic parameters (*n*, ∆*G*, ∆*H*, ∆*S*) of the interaction between human serum albumin and extracts from red and yellow fruits of *Cornus mas* L. at five temperatures. Standard deviations (mean value of three independent experiments) were lower than 10%.

Compound	*T* (*K*)	*K_SV_* (mL/g)	*K_b_* (mL/g)	*n*	∆*G* (kJ/g·mL^−1^)	∆*H* (kJ/g·mL^−1^)	∆*S* J/(g·mL^−1^·K)
RC	295	30.91 × 10^3^	3.705 × 10^4^	1.05	−25.72	−24.88	30.04
	300	29.51 × 10^3^	2.985 × 10^4^	0.99	−25.67		
	305	28.78 × 10^3^	2.505 × 10^4^	0.93	−25.64		
	310	27.82 × 10^3^	2.128 × 10^4^	0.87	−25.63		
	315	27.04 × 10^3^	1.933 × 10^4^	0.83	−25.81		
YC	295	16.52 × 10^3^	1.752 × 10^4^	1.09	−23.95	−21.72	11.17
	300	13.59 × 10^3^	1.407 × 10^4^	1.01	−23.75		
	305	12.22 × 10^3^	1.277 × 10^4^	0.97	−23.87		
	310	11.52 × 10^3^	1.001 × 10^4^	0.92	−23.72		
	315	10.85 × 10^3^	0.819 × 10^4^	0.85	−23.60		

## Data Availability

Data is contained within the article.

## Sample Availability

Samples of the compound red and yellow of *Cornus mas* L. extracts are available from the authors.
